# 2376: Best practices for social and behavioral research: Developing a competency-based elearning course in good clinical practice

**DOI:** 10.1017/cts.2017.175

**Published:** 2018-05-10

**Authors:** Susan Lynn Murphy, Christy Byks-Jazayeri, Elizabeth Anderson, Angela Lyden, Jennifer Miner, Jordan Hahn, Brandon Lynn

**Affiliations:** University of Michigan School of Medicine, Ann Arbor, MI, USA

## Abstract

OBJECTIVES/SPECIFIC AIMS: Existing GCP training is geared primarily towards researchers conducting drug, device, or biologic clinical trials, and largely ignores the unique needs of researchers conducting social and behavioral clinical trials. The purpose of this project was to develop a comprehensive, relevant, interactive, and easy to administer GCP eLearning course for social and behavioral researchers. METHODS/STUDY POPULATION: As part of the ECRPTQ project funded by the National Center for Advancing Translational Sciences (NCATS), a Social and Behavioral Work Group of ~30 experienced social and behavioral investigators and study coordinators was formed to develop GCP training for social and behavioral researchers. Existing GCP training programs were reviewed to identify relevant content that should be included as well as gaps specific to social and behavioral clinical trials where new content would need to be developed. In total, 9 specific modules—Introduction, Research Protocol, Roles and Responsibilities, Informed Consent Communication, Confidentiality/Privacy, Recruitment/Retention, Participant Safety/Adverse Event Reporting, Quality Control/Assurance, and Research Misconduct—were identified by the work group and the content was mapped to competency domains defined by the ECRPTQ project, as well as International Council for Harmonisation (ICH) GCP principles. Several investigators and study coordinators were identified as content experts for each module topic. Working with an instructional designer, these experts defined learning objectives and outlined content relevant for both study coordinators and investigators for inclusion in the modules. The curriculum was developed using Articulate Storyline that is SCORM 1.2 compliant making the course usable to the widest audience. The course was designed to be administered on laptop or desktop computers and is accessible for individuals with hearing or viewing impairments. To maximize learning, instructional designers used creative treatments including: narration to guide learners or offer tips; short video scenarios to introduce topics; interactive activities, such as drag and drop games and “click to learn more information”; knowledge checks with feedback; resources, including downloadable job aids; end of module quizzes, and documentation of course completion. The full curriculum takes 2–4 hours to complete, with individual modules taking 30 minutes to complete. RESULTS/ANTICIPATED RESULTS: Pilot testing to evaluate the effectiveness of the eLearning course is underway at 5 sites: University of Michigan, Boston University, University of Rochester, University of Florida, and SUNY Buffalo. DISCUSSION/SIGNIFICANCE OF IMPACT: This eLearning course provides relevant, comprehensive GCP training specifically for social and behavioral researchers. Unlike existing GCP training that is geared towards drug and device researchers, this course includes scenarios and examples that are relevant to social and behavioral researchers. The engaging, interactive nature of this course is designed to improve learning and retention, resulting in improved job performance. In addition, the modules are designed for both investigators and clinical research coordinators, thus eliminating the need for different training modules for different study team members.

